# Supplementary material for: Gradual Stress-Relaxation of Hydrogel Regulates Cell Spreading
Source: Int J Mol Sci. 2022 May 5;23(9):5170. doi: 10.3390/ijms23095170 (PMC9100461; doi:10.3390/ijms23095170)
Supplement: Supplementary file 1 [file ijms-23-05170-s001.zip › ijms-1703163 - supp.pdf]

International Journal of
Molecular Sciences

MDPI

Supplementary Information

Gradual Stress-relaxation of Hydrogel Regulates Cell Spreading

Wenting Yu [1,†], Wenxu Sun [3], Huiyan Chen [2], Juan Wang [2], Bin Xue [2,*], and Yi Cao [1,2,*]

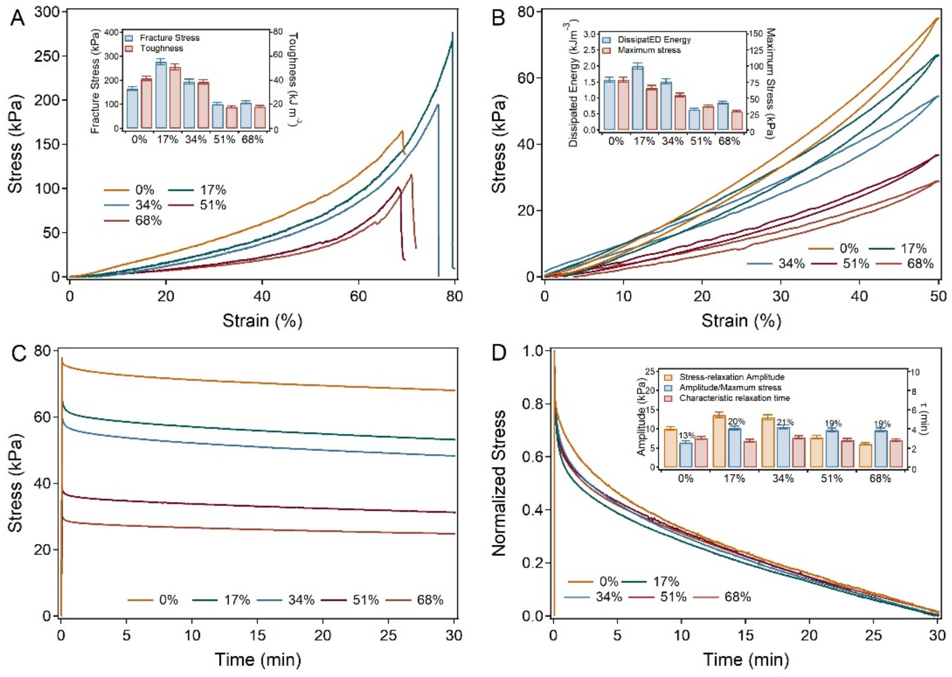

Figure S1. The mechanical properties of the band-like hydrogel without coordination, similar locations to those of SRG hydrogels were presented (0%, 17%, 34%, 51% and 68%). (A) Compression-cracks of the band-like hydrogel. Summarized fracture stress and toughness corresponding to different locations on the band-like hydrogel (insert). (B) Compression-relaxation cycles of different locations on the band-like hydrogel. Summarized dissipated energy and maximum stress corresponding to different locations on the band-like hydrogel (insert). Compression stress-relaxation curve (C) and normalized compression stress-relaxation curve (D) of different locations on the hydrogel over 30 minutes. Summarized stress-relaxation amplitudes and characteristic relaxation time (τ) at different locations of the band-like hydrogel (insert)

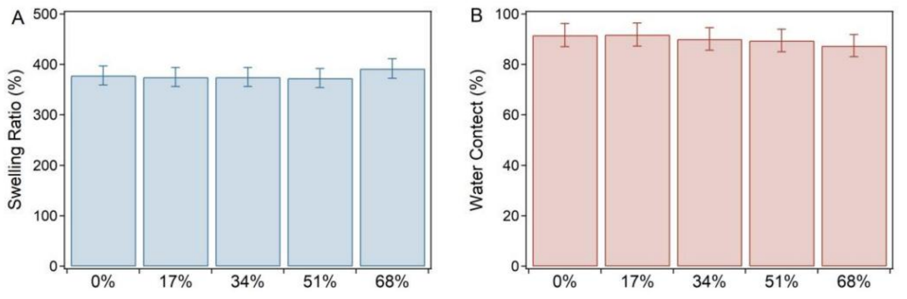

Figure S2. Swelling ratios (A) and water contents (B) corresponding to different locations on the SRG hydrogel.

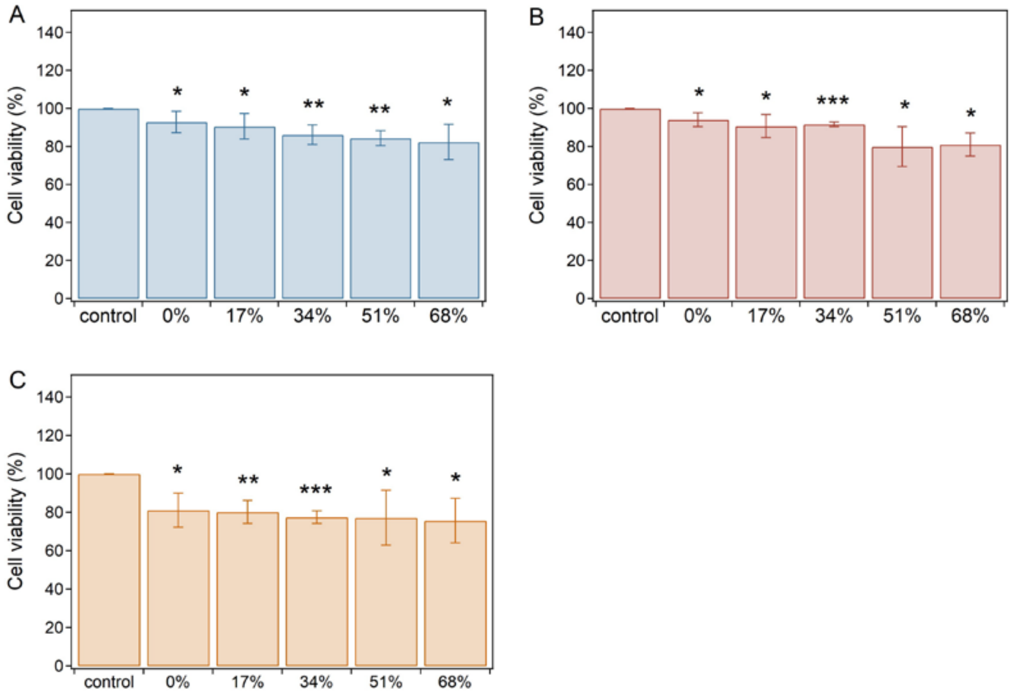

Figure S3. Cell viabilities of L929 (A), HeLa (B), and primary mouse cardiac fibroblasts (C) cells cultured on SRG hydrogels after being cultured for 24 hours. The cells cultured on wells were set as the 100% groups. Asterisks denote statistical significance compared with control (P>0.05: N.S.; P < 0.05: *; P < 0.01: **; P < 0.001: ***)

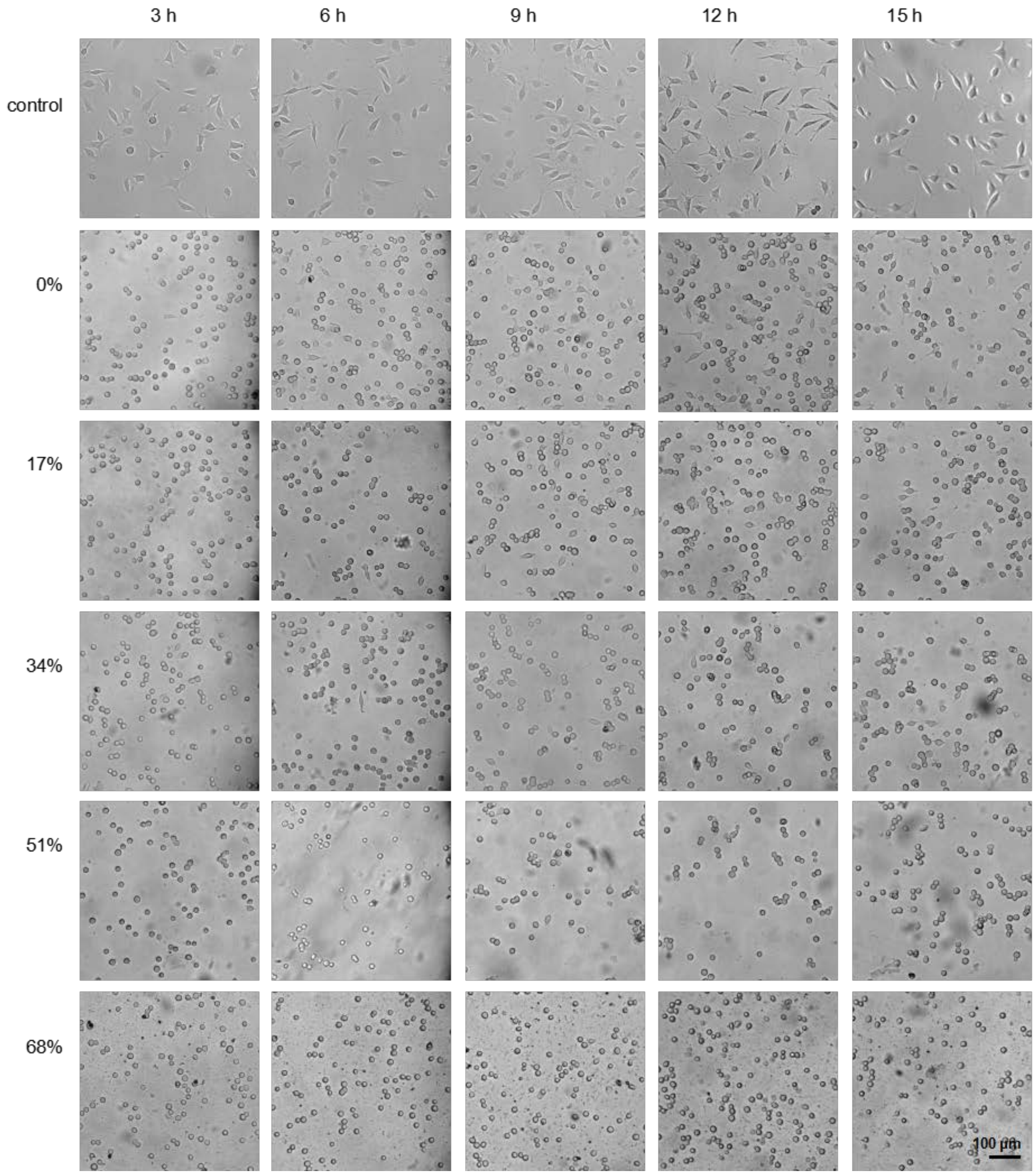

Figure S4. Representative images of L929 cells spreading on different locations of the SRG hydrogels after being cultured for different times.

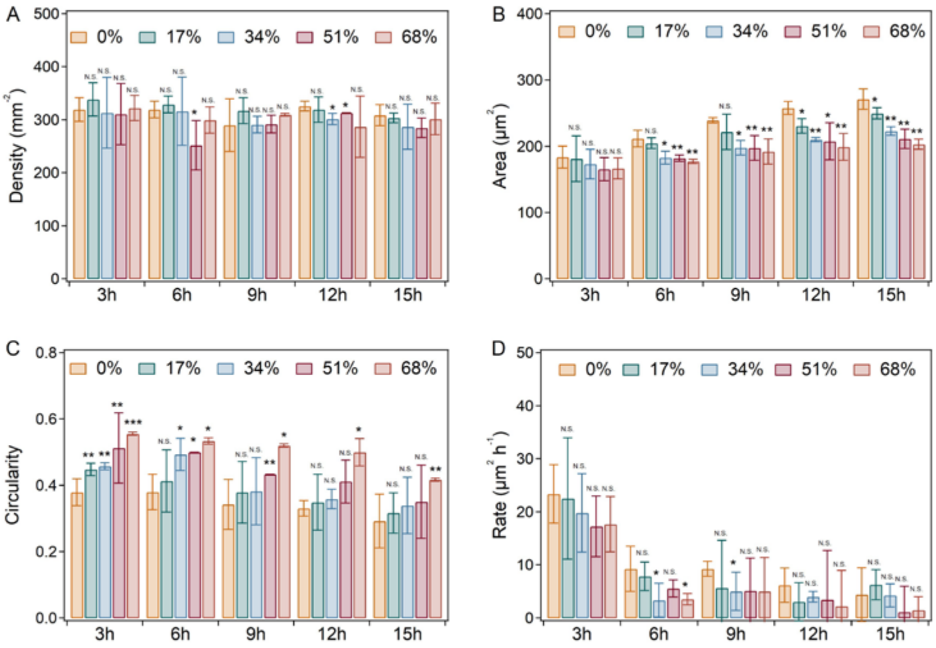

Figure S5. Summary of L929 cells spreading on SRG hydrogels. Cell density (A), cell-spreading area (B), cell circularity (C) and cell-spreading rate (D) of L929 cells on SRG hydrogels. Asterisks denote statistical significance compared with 0% location at the same time (P>0.05: N.S.; P < 0.05: *; P < 0.01: **; P < 0.001: ***).

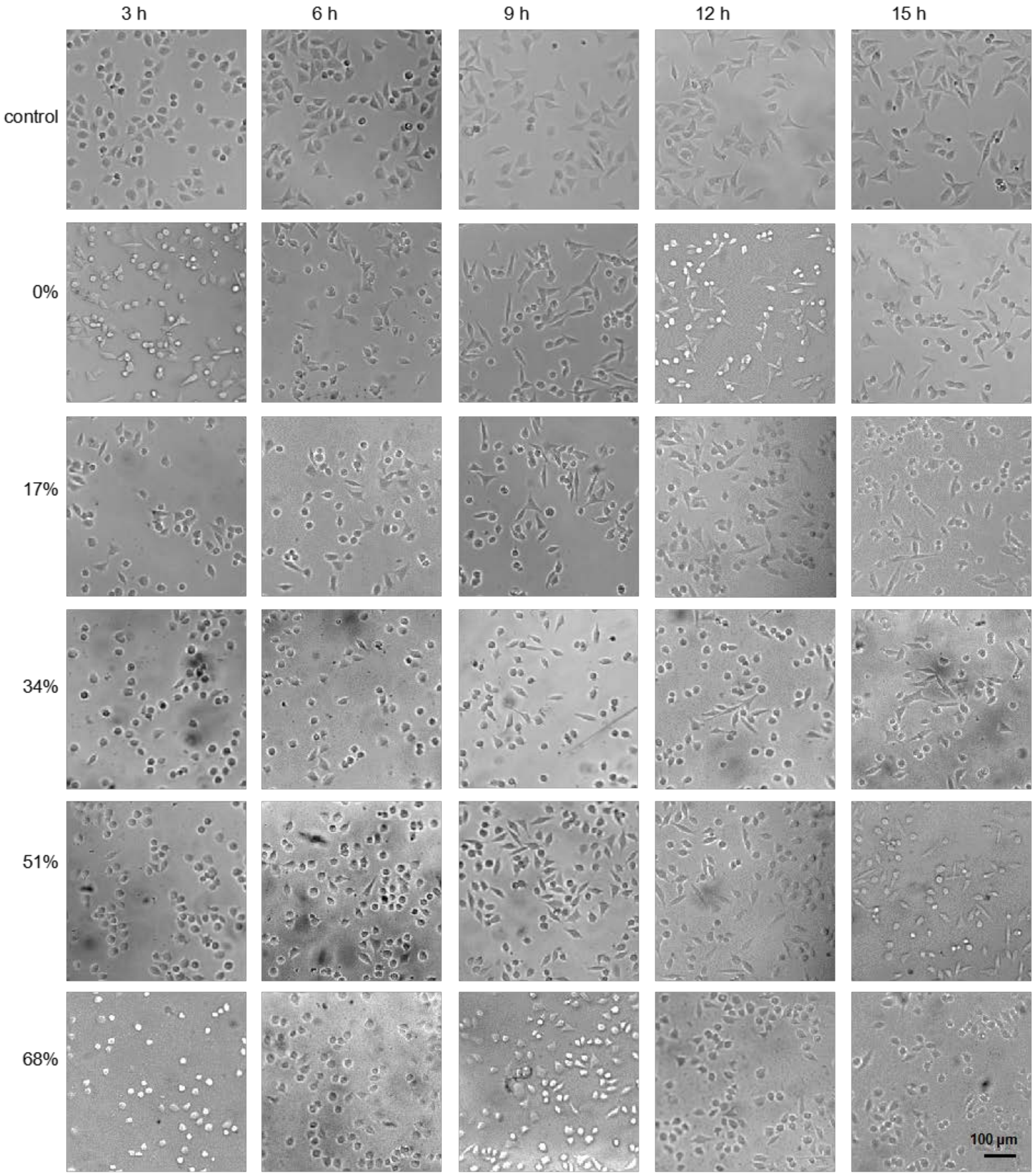

Figure S6. Representative images of HeLa cells spreading on different locations of the SRG hydrogels after being cultured for different times.

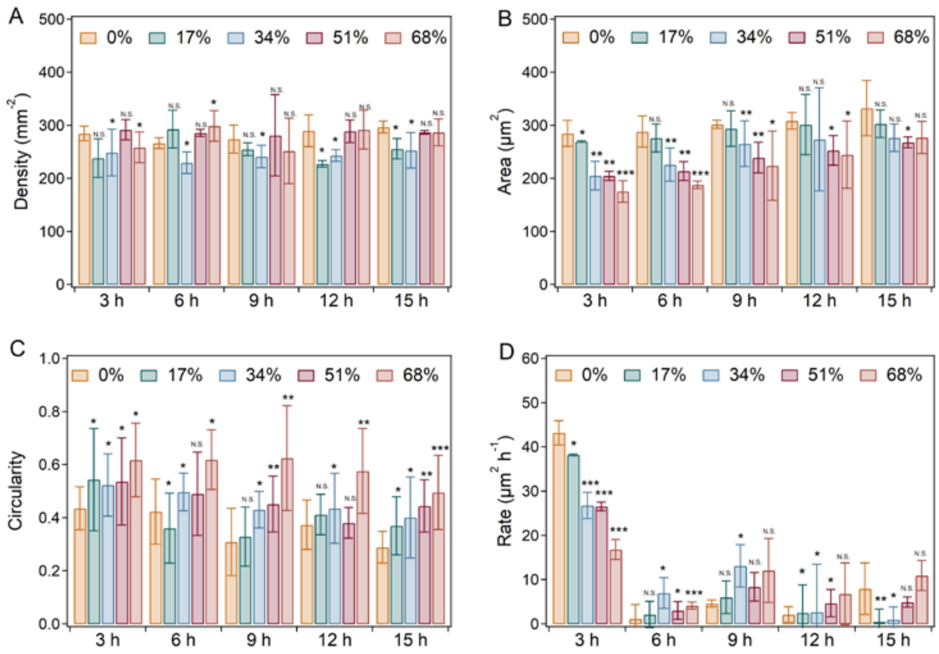

Figure S7. Summary of HeLa cells spreading on SRG hydrogels. Cell density (A), cell-spreading area (B), cell circularity (C) and cell-spreading rate (D) of HeLa cells on SRG hydrogels. Asterisks denote statistical significance compared with 0% location at the same time (P>0.05: N.S.; P < 0.05: *; P < 0.01: **; P < 0.001: ***).

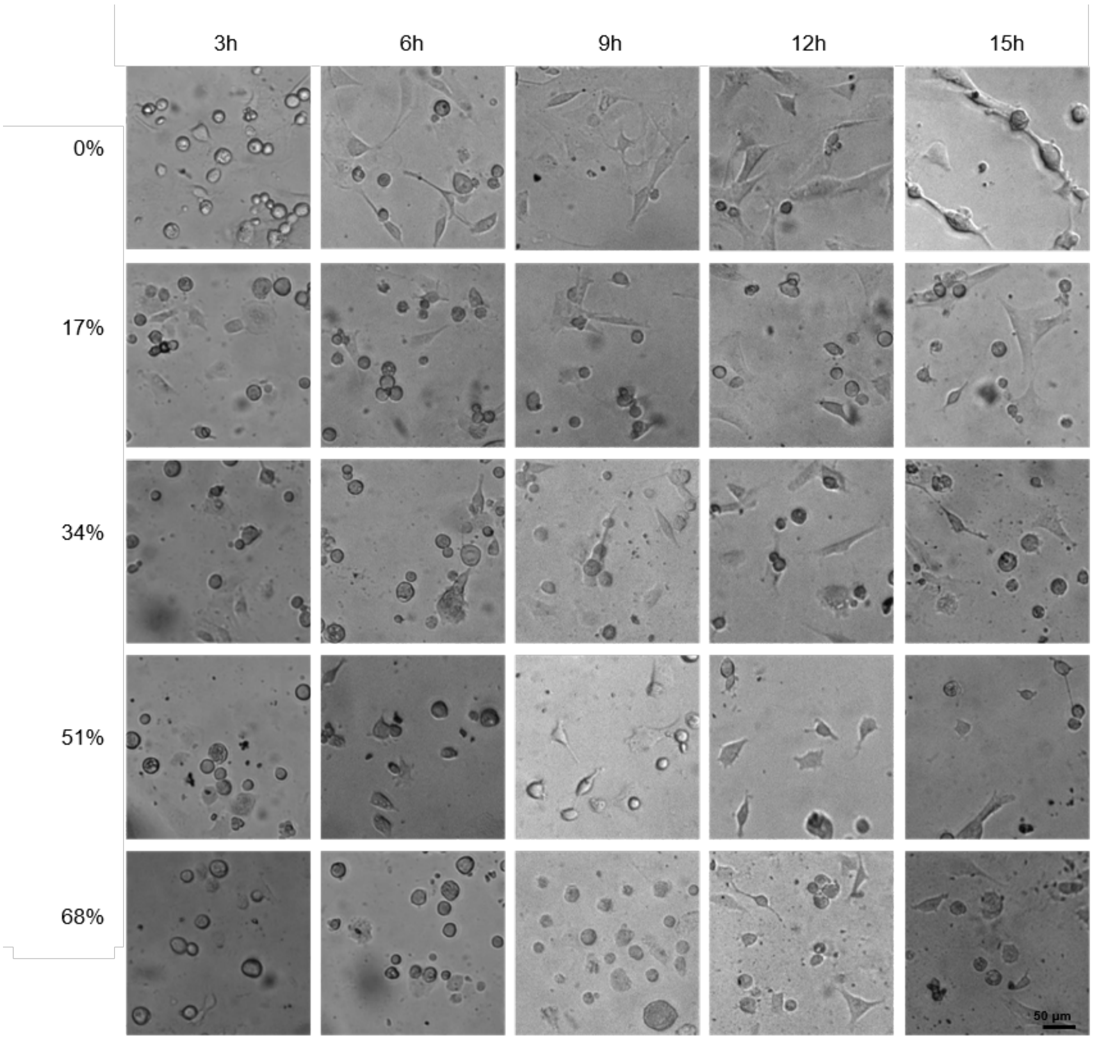

Figure S8. Representative images of MCFs spreading on different locations of the SRG hydrogels after being cultured for different times.

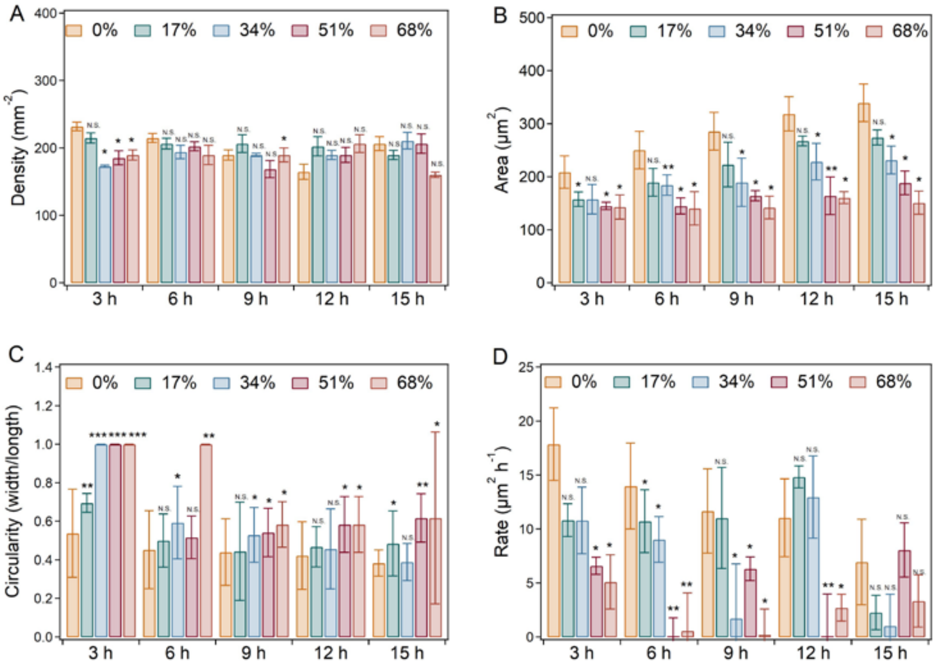

Figure S9. Summary of MCFs spreading on SRG hydrogels. Cell density (A), cell-spreading area (B), cell circularity (C) and cell-spreading rate (D) of MCFs on SRG hydrogels. Asterisks denote statistical significance compared with 0% location at the same time (P>0.05: N.S.; P < 0.05: *; P < 0.01: **; P < 0.001: ***).

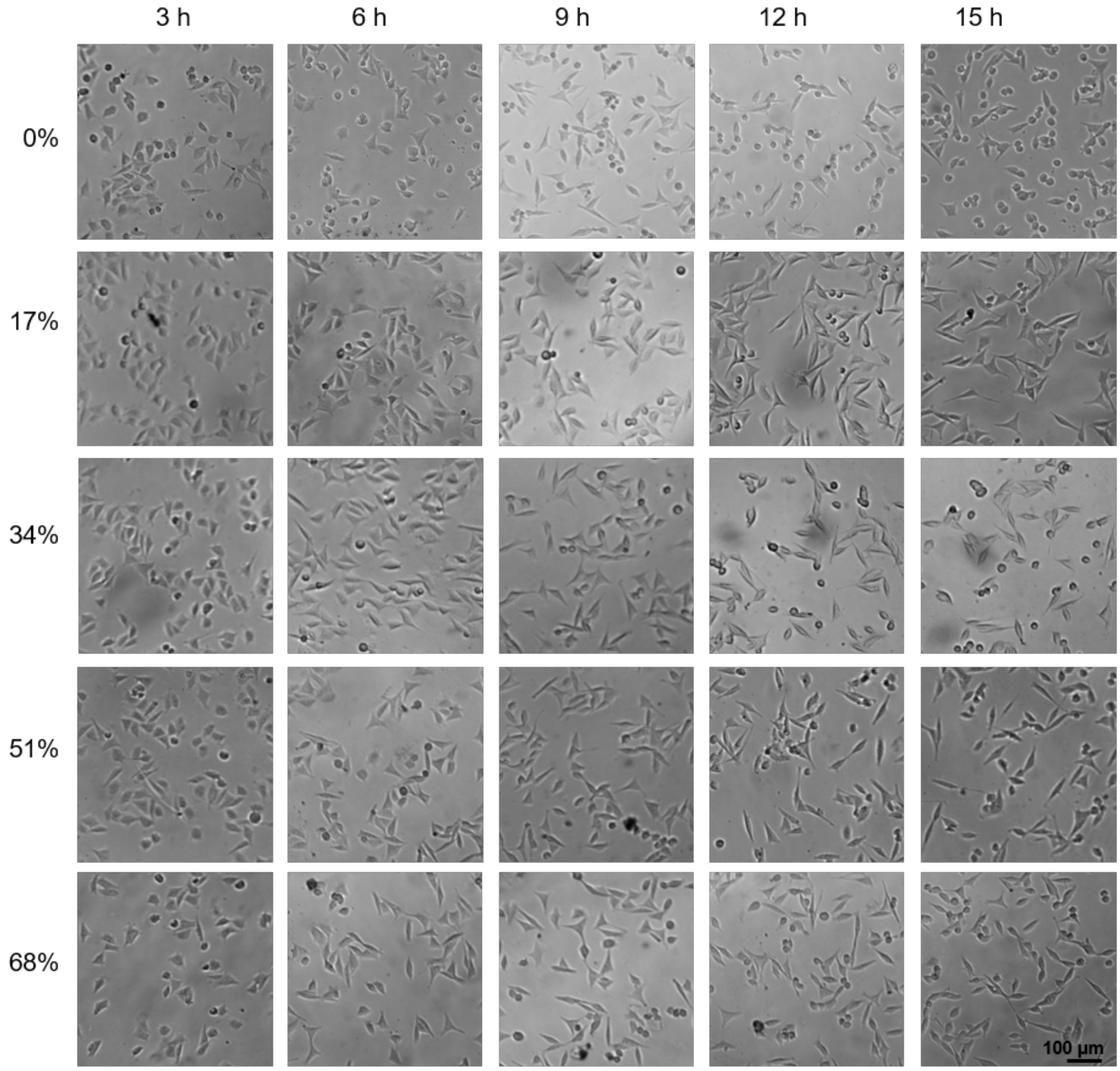

Figure S10. HeLa cells spreading on the band-like hydrogel without coordination. Similar locations to those of SRG hydrogels were presented (0% location, 17% location, 34% location, 51% location and 68% location). Hydrogels with elasticity gradients were prepared using the same method as SRG hydrogels without PH_6-Zn^{2+} crosslinkers.

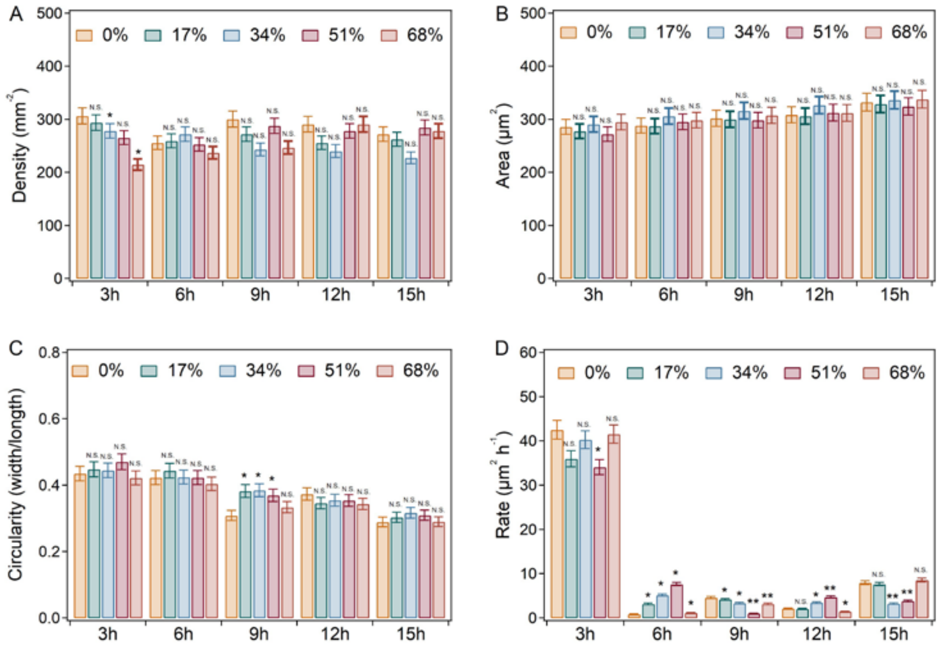

Figure S11. Summary of HeLa cells spreading on band-like hydrogels without coordination. Cell density (A), cell-spreading area (B), cell circularity (C) and cell-spreading rate (D) of HeLa cells on SRG hydrogels. Asterisks denote statistical significance compared with 0% location at the same time (P>0.05: N.S.; P < 0.05: *; P < 0.01: **; P < 0.001: ***).

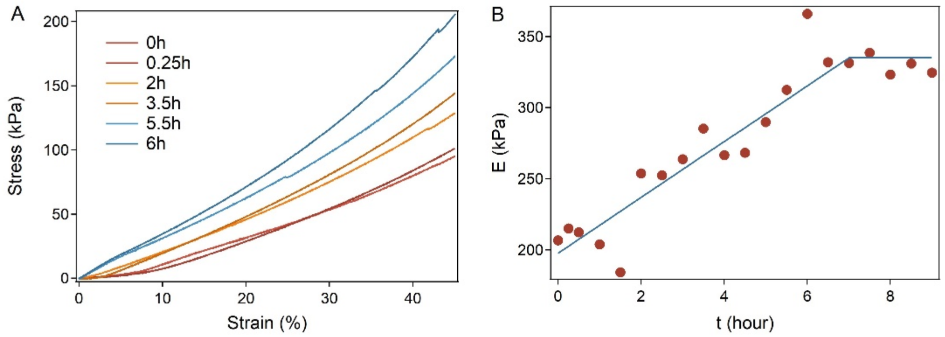

Figure S12. Young's modulus of hydrogels after being immersed in Zn^{2+} solutions for different times. (A) Typical stress-strain curves of hydrogels after being immersed in Zn^{2+} solutions for different times. (B) Summarized Young's modulus (E) for hydrogels after being immersed for different times (t). The blue line indicates the linear fitting.

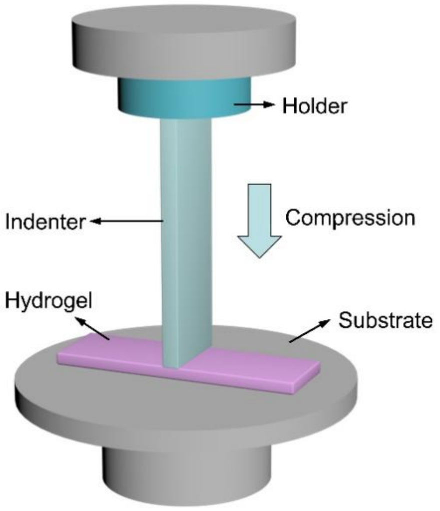

Figure S13. Illustration of the mechanical measurements of SRG hydrogels. The indenter of a compressive tester used to compress different locations of a band-shaped hydrogel and the force-distance curves were recorded.
